# Utility of a novel point-of-care test in detecting coronary artery disease following negative nuclear testing: a case series

**DOI:** 10.1093/ehjcr/ytag016

**Published:** 2026-01-23

**Authors:** Mustafa Alkhawam, Timothy Burton, Horace R Gillins, Cody Harris, Shyam Ramchandani, Charles R Bridges, Parminder B Singh, Tracy Neal

**Affiliations:** Division of Cardiovascular Disease, The University of Alabama at Birmingham, 1720 2nd Ave South Birmingham, AL 35294, USA; Analytics for Life, 100 King St W Suite 5600, Toronto, ON M5X 1C9, Canada; CorVista Health, 3 Bethesda Metro Center, Suite 700, Bethesda, MD 20814, USA; Cullman Regional Medical Group, 1912 Alabama Highway 157, Ground Floor, Main Hospital, Cullman, AL 35058, USA; Analytics for Life, 100 King St W Suite 5600, Toronto, ON M5X 1C9, Canada; CorVista Health, 3 Bethesda Metro Center, Suite 700, Bethesda, MD 20814, USA; Marion General Hospital, 1000 McKinley Park Dr, Marion, OH 43302, USA; Cullman Regional Medical Group, 1912 Alabama Highway 157, Ground Floor, Main Hospital, Cullman, AL 35058, USA

**Keywords:** Non-invasive, SPECT, Ischaemia, Angiography, Machine Learning

## Abstract

**Background:**

Coronary artery disease (CAD) often presents diagnostic challenges, especially in patients with atypical chest pain that persists despite negative non-invasive testing. For instance, nuclear stress testing, although widely used, may yield false-negative results, particularly in cases of balanced ischaemia. Accurate diagnostics are essential to avoid delays in diagnosis and treatment, particularly in the presence of negative nuclear testing.

**Case summary:**

We present five cases of intermediate- to high-risk patients with ongoing symptoms (chest pressure/discomfort/pain, nausea, shortness of breath, and exertional fatigue) and negative nuclear stress tests, who were ultimately diagnosed with significant CAD via invasive coronary angiography. In each case, CorVista CAD, a novel point-of-care test for CAD (POC-CAD) that analyses electrical and haemodynamic signals using machine learning, was contributory in raising the probability of disease, prompting invasive evaluation via coronary angiography and guiding appropriate management strategies.

**Discussion:**

This case series demonstrates the utility of POC-CAD as a non-invasive tool in the diagnostic pathway for patients with ongoing symptoms despite negative nuclear stress testing. The findings highlight limitations of nuclear stress testing, particularly its reduced sensitivity in multi-vessel disease. POC-CAD may help identify high-risk patients who would otherwise be missed, facilitating timely intervention. Integration of novel diagnostics such as POC-CAD into multimodal assessment strategies could improve accuracy and reduce diagnostic delays in CAD.

Learning points

- Negative nuclear stress testing often does not exclude significant coronary artery disease (CAD), particularly in patients with persistent symptoms.
- A novel point-of-care test for CAD may aid in identifying high-risk patients missed by traditional modalities.
- Early and accurate diagnosis facilitated by advanced diagnostics can lead to improved patient outcomes through timely intervention.

## Introduction

Coronary artery disease (CAD) remains a leading cause of morbidity and mortality worldwide, yet its diagnosis can be particularly challenging in patients with persistent symptoms and negative non-invasive test results. While nuclear stress testing, i.e. single photon emission computed tomography (SPECT), plays a central role in evaluating ischaemia, particularly in the USA, false negatives may occur, particularly in cases of balanced ischaemia. This case series highlights five patients whose CAD was identified despite negative nuclear stress testing. The introduction of CorVista CAD (Analytics for Life & CorVista Health), a novel point-of-care test for CAD (POC-CAD), provided additional diagnostic insight in these five intermediate- to high-risk patients, increasing the probability of disease and prompting further evaluation via invasive coronary angiography (ICA).

POC-CAD noninvasively records orthogonal voltage gradient (via seven thorax electrodes) and photoplethysmogram (via a fingerclip sensor) signals from a resting patient using a portable device over 3.5 min. The signals are transmitted to a secure cloud platform where a machine-learning algorithm analyses features derived from the signals to generate a score between −0.5 and +0.5. Therefore, POC-CAD can be deployed anywhere with an Internet connection and does not require capital cost expenditure nor specialized training, thus facilitating access in underserved areas (e.g. rural, where travel to the nearest imaging centre is often substantial).

POC-CAD was trained (*N* = 1154) on a cohort representative of the clinical population, composed of subjects confirmed to have or not have significant CAD via gold standard ICA based on the American College of Cardiology (ACC) guidelines.^1, 2^ Further negative subjects used for training were ruled out for significant CAD via coronary computed tomography angiography (CCTA), which has excellent negative predictive value for absence of disease.^3^ POC-CAD was validated in a separate, blinded, enrolment-gated cohort of *N* = 1816, yielding sensitivity of 88% and specificity of 51%. Scores ≥ 0 indicate ‘possibly significant CAD’, prompting further evaluation, while scores < 0 rule out significant disease with 99% negative predictive value given a disease prevalence of 4% (as derived from the PROMISE study^4^). At the same 4% prevalence, treated as the pre-test probability, the positive predictive value is 7%; while that appears modest (not atypical for a rule-out test), the test can have increased ability to detect disease with two mechanisms: firstly, many patients have a pre-test probability higher than that of the disease prevalence in PROMISE (e.g. all the cases discussed in the present series), and secondly, likelihood ratios increase with higher test scores.^5^

POC-CAD is now Food and Drug Administration (FDA) cleared and commercially available in the USA to indicate the likelihood of CAD in patients with stable cardiovascular symptoms. The POC-CAD is not indicated in patients with symptoms consistent with an acute coronary syndrome (ACS), since these patients were excluded from the blinded prospective validation study.^1^ The POC-CAD output is presented for interpretation by healthcare providers in conjunction with their clinical judgement and the patient’s signs, symptoms, and clinical history as an aid of diagnosis; therefore, POC-CAD does not independently dictate care, limiting risk associated with false-positive and false-negative results. However, in isolation, a false negative may delay required testing (infrequent given sensitivity of 88%), while a false positive tends to lead to additional unneeded testing (more frequent given specificity of 51%). To assess the economic impact of POC-CAD given the test performance characteristics, POC-CAD was positioned in a diagnostic pipeline with positron emission tomography (PET), such that every POC-CAD test-positive patient received a PET, and when positive, referred to ICA.^5^ Considering the costs for POC-CAD, PET, and ICA, along with the cost of a false negative that arrives at the ER with ACS, the cost per patient of this proposed diagnostic pipeline is $4510 per patient. In contrast, a similar pipeline involving SPECT and ICA yielded an average cost per patient of $5682, an increase of $1172, which is largely attributable to patients lost to follow-up (e.g. due to inability to travel to a centre that offers SPECT) that subsequently present acutely at the ER.

This series illustrates the utility of POC-CAD as a complementary tool in clinical decision-making in intermediate- to high-risk patients, particularly those with persistent symptoms in the presence of negative or equivocal SPECT imaging.

## Summary figure

**Figure:**
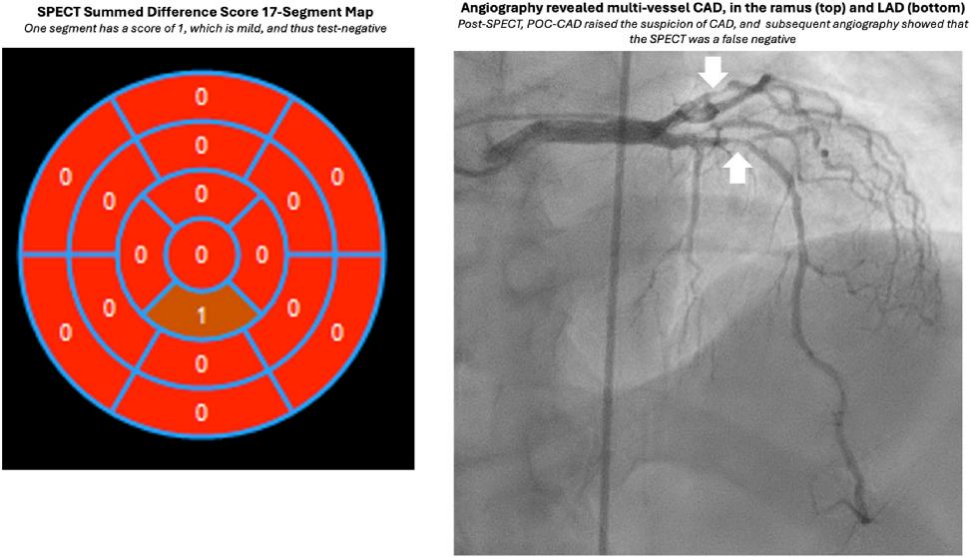


## Case presentations

The patients’ pre-test probabilities (PTPs) for CAD are shown in *Table 1*.

**Table 1:** Pre-test probability of coronary artery disease using ACC, European Society of Cardiology (ESC), and risk-factor-weighted models, based on demographics and symptoms (excluding any test results)

	Relevant characteristics	ACC^6^	ESC^7^	Risk factor weighted^8^
Case 1	63-year-old male; hyperlipidaemia, family history of cardiovascular disease, past tobacco use; typical angina	<44%	44%	35%
Case 2	72-year-old female; diabetes, hypertension; typical angina	<27%	27%	19%
Case 3	81-year-old male; hyperlipidaemia, hypertension; atypical angina	<52%	34%	27%
Case 4	82-year-old female; dyslipidaemia, hypertension; atypical angina	<27%	19%	10%
Case 5	67-year-old female; hypertension, hyperlipidaemia, diabetes, family history of cardiovascular disease; atypical angina	<16%	11%	11%

The cases typically fall well into the diagnostic range of >15% where further investigation is warranted.

**Figure 1:**
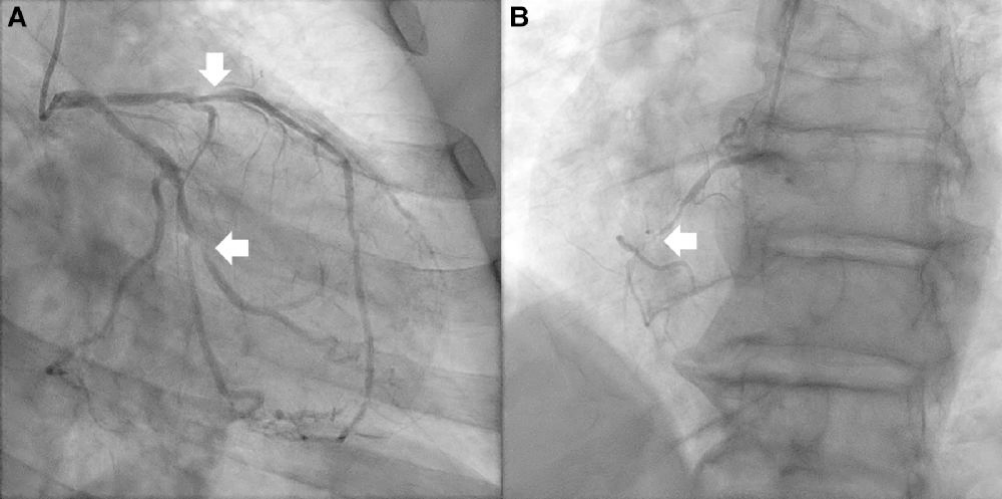
Case 1 diagnostic angiography, showing, (*A*) at the top arrow, the 80% left anterior descending artery lesion, and at the bottom arrow, the left circumflex mid-segment (80%) and second marginal lesions (80%). See Supplementary material online, *Video S1*. In (*B*), the collateralized subtotally occluded right coronary artery is visible (Supplementary material online, *Video S2*).

**Figure 2:**
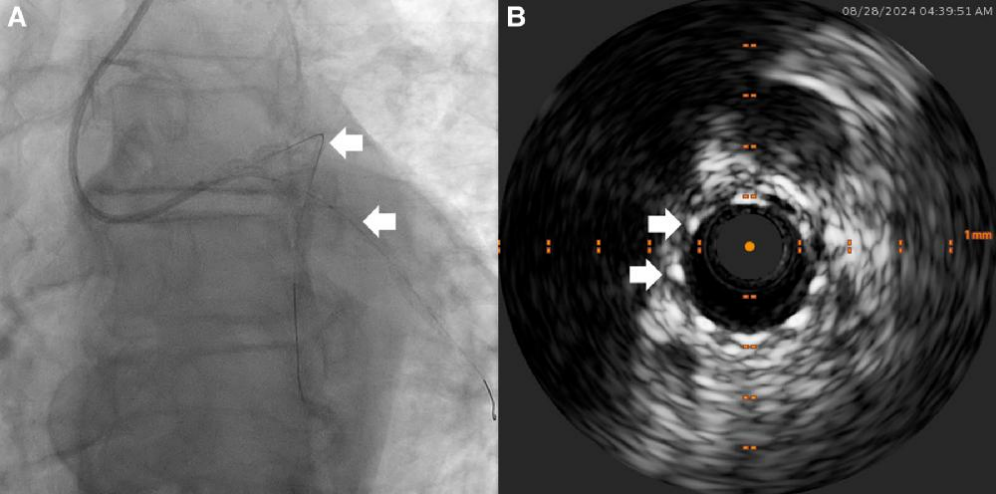
Intervention for Case 1, showing (*A*) the use of multiple guidewires (marked by arrows) to place the stents in the left circumflex and (*B*) intravascular ultrasound confirmation of stent placement in the left circumflex, with example stent struts identified by arrows (Supplementary material online, *Video S3*).

**Case 1:** A 63-year-old male with a body mass index (BMI) of 26 presented with progressively increasing chest pain and shortness of breath on exertion. He had a history of hyperlipidaemia, prediabetes, past tobacco use, and a significant family history of cardiovascular disease. His chest pain was initiated by strenuous activity, resolved within 30 s–2 min with rest, and was not associated with jaw or left arm discomfort, meeting criteria for typical angina. He had ceased tobacco use recently and consumes alcohol occasionally. Despite undergoing extensive testing over the past 5 years, including a normal transthoracic echocardiogram (TTE) in 2019, a negative nuclear stress test in 2022, and a 7-day Holter monitor study in 2023 showing low premature ventricular contraction (PVC) and premature atrial contraction (PAC) burdens, the aetiology of his symptoms remained unclear. Given his ongoing symptoms and a positive POC-CAD result (score of 0.20), he underwent ICA (2 weeks post-POC-CAD), which revealed severe multi-vessel CAD (*Figure 1*). The left anterior descending artery (LAD) exhibited 80% stenosis from the ostial to mid-segment, the first diagonal (D1) was 60% stenosed, and the left circumflex (LCX) showed 75% stenosis from the ostial to proximal segment. The mid-segment of the LCX was 80% stenosed, and the mid- to distal LCX had 60% stenosis. The LCX second marginal (M2) had 80% stenosis. Additionally, the right coronary artery (RCA) was subtotal occluded but collateralized from the LCX. The patient underwent complex percutaneous coronary intervention (PCI) (*Figure 2*) 2 days post-ICA with four stents placed at significant stenotic lesions in the LAD and LCX, which resulted in marked symptomatic improvement, and allowed the patient to return to active employment in construction.

**Figure 3:**
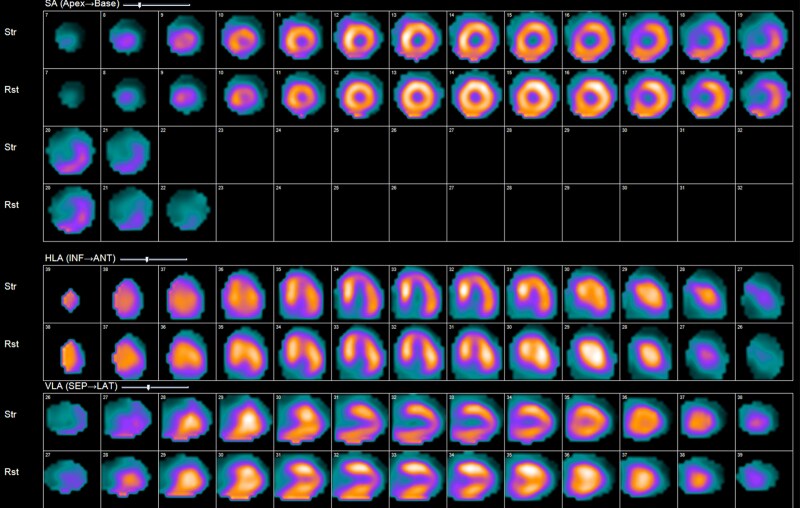
Slice-level images from the Case 2 single photon emission computed tomography.

**Figure 4:**
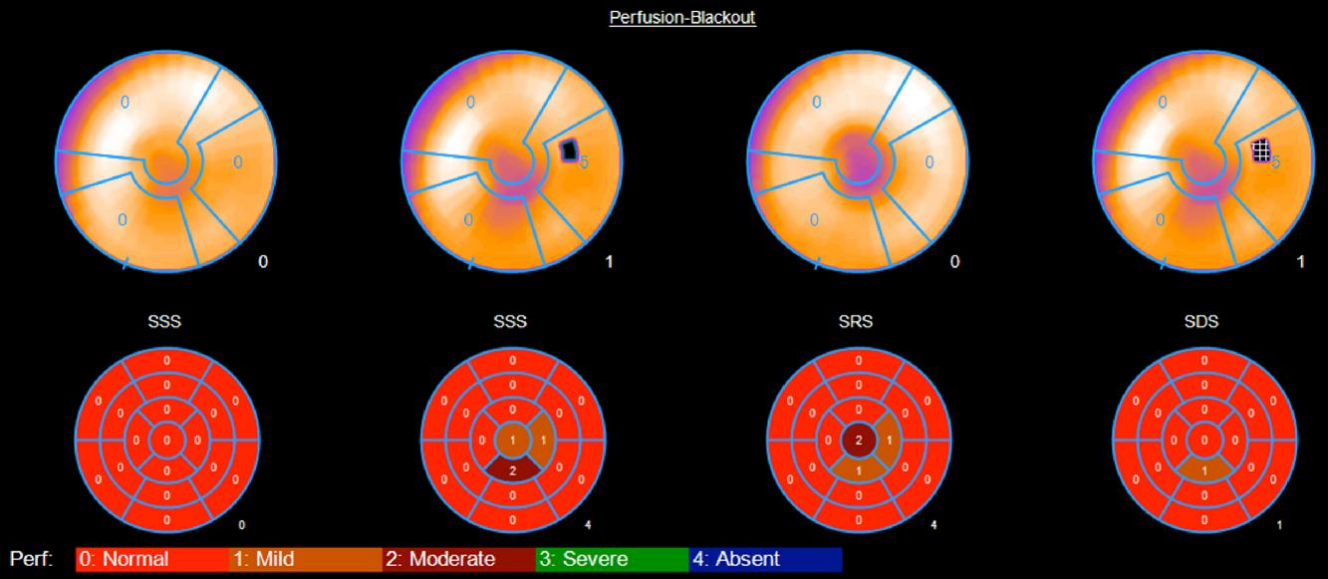
Single photon emission computed tomography 17-segment model for Case 2, showing no significant ischaemia and thus test-negative.

**Case 2:** A 72-year-old female with Type II diabetes and hypertension experienced daily episodes of chest pressure, lasting anywhere from 5 to 30 min, with exertional fatigue and shortness of breath both relieved with rest (qualifying as typical angina). She did not have hyperlipidaemia, nor any history of tobacco use, nor a family history of cardiovascular disease. A nuclear stress test was administered 2 months prior, in which she had difficulty achieving target heart rate on treadmill testing, and thus was given Lexiscan. The pharmaceutical heart rate elevation resulted in chest pain, shortness of breath, and nausea, accompanied by non-specific ST changes. The imaging showed a mild apical defect on the stress images with no evidence of ischaemia on rest imaging and was attributed to soft tissue attenuation (*Figures 3* and *4*). Therefore, given the non-specific ST changes, the nuclear stress test was concluded to be ‘clinically positive’ only, with no detectible ischaemia on imaging. Despite this result, the symptoms persisted for another 2 months, and a subsequent calcium scan revealed a score exceeding 700. POCCAD was administered, yielding a positive score of 0.11. Based on the ongoing symptoms, in the setting of abnormal POC-CAD and the calcium score, balanced ischaemia was suspected. Subsequent ICA identified critical stenoses: 99% in the mid-LAD, 90% at the ostium of the D1 branch, and 70% in the ramus intermedius. Ventriculography revealed an ejection fraction (EF) of 60%, with left ventricular end-diastolic pressure (LVEDP) of 12 mmHg. Percutaneous coronary intervention was recommended.

**Figure 5:**
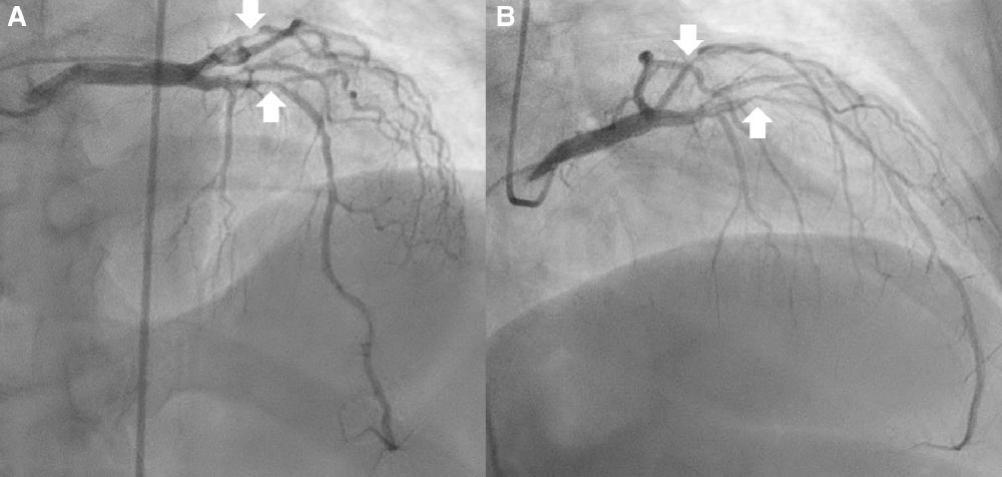
Diagnostic angiography for Case 2, showing two views of the 70% ramus left (top arrow) and 99% left anterior descending artery lesion (bottom arrow). Supplementary material online, *Video S4* further illustrates (*A*) and Supplementary material online, *Video S5*, (*B*).

**Figure 6:**
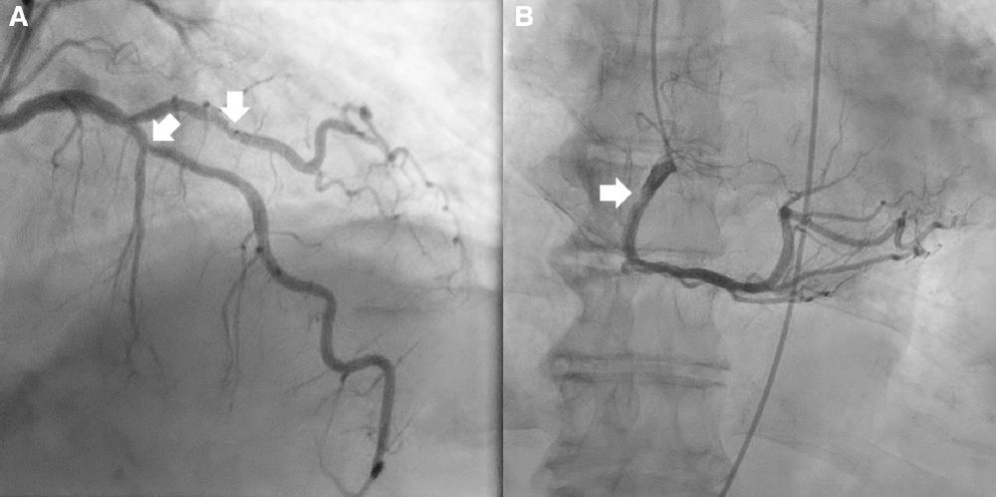
Diagnostic angiography for Case 3, showing (*A*) the 60%–70% left anterior descending artery first diagonal lesion at the upper right arrow and the 72% left anterior descending artery lesion at the bottom left arrow (Supplementary material online, *Video S6*). In (*B*), the 30%–40% right coronary artery lesion is visible (Supplementary material online, *Video S7*).

**Case 3:** An 81-year-old male with hyperlipidaemia and hypertension presented with atypical chest discomfort (ache around the left chest), not consistently related to exertion. He had no history of diabetes or smoking. The patient had a nuclear stress test almost 2 years prior and, during the baseline rest portion, exhibited a normal ECG in sinus rhythm and a blood pressure of 194/108 mmHg. The patient was unable to reach target heart rate via exercise, and thus Lexiscan was administered, causing a heart rate elevation to 125 beats per minute and a peak blood pressure of 232/105 mmHg. However, there was no resultant chest pain, nor ST changes. Finally, the stress images showed no signs of ischaemia. A recent calcium scan revealed a calcium score of 974, predominantly in the LAD. POCCAD was positive, leading to the suspicion of balanced ischaemia and thus ICA. The angiography yielded a 72% stenosis in the mid-LAD by quantitative coronary angiography (QCA) distal to the D1, 50%–60% ostial stenosis in the D1 branch, 60%–70% lesion in the mid-D1, and 30–40% diffuse disease in the mid-RCA (*Figure 6*). Left ventricular end-diastolic pressure was measured at 12 mmHg, and EF was 60%. Given the patient’s age, a conservative approach of initiating medical therapy was pursued, but if symptoms persist, then catheterization will be repeated with intravascular ultrasound (IVUS) and fractional flow reserve (FFR) to further assess the haemodynamic significance of the mid-LAD lesion with consideration of PCI if indicated.

**Figure 7:**
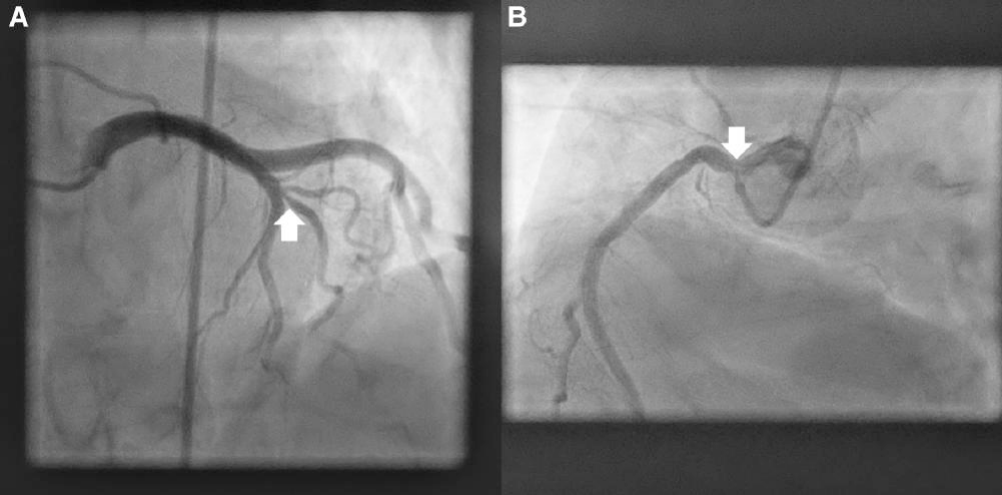
Diagnostic angiography for Case 4, showing (*A*) the 75.1% left anterior descending artery first diagonal lesion by quantitative coronary angiography (Supplementary material online, *Video S8*) and (*B*) the 39% ostial right coronary artery lesion by quantitative coronary angiography (Supplementary material online, *Video S9*).

**Case 4:** An 82-year-old female with dyslipidaemia, hypertension, and paroxysmal atrial fibrillation reported progressively worsening atypical sensation across her chest and intermittent nausea. She also reported significant exertional shortness of breath, consistent with atypical angina. The patient underwent a nuclear stress test approximately a year ago, where the baseline ECG at rest was normal, with no ST abnormalities. Lexiscan was administered to induce stress, yielding no perfusion defects. Given the persistence of symptoms, POC-CAD was administered, which was test-positive. Given the positive POC-CAD, as well as the significant ongoing symptoms, significant CAD was suspected, and thus ICA was performed. Angiography found luminal irregularities throughout the LAD system, with a 75.1% stenosis in the D1 branch by QCA. Further, there was a 39% lesion (by QCA) in the ostial RCA, with a 20 mmHg gradient across that lesion (*Figure 7*). Medical therapy was recommended, as an intervention on the D1 lesion would potentially compromise the main LAD channel. Approximately 7 months after angiography, the patient has performed well on spironolactone and sacubitril/valsartan (combination therapy), and therefore neither further invasive investigation nor intervention is warranted at this time.

**Case 5:** A 67-year-old morbidly obese (BMI 41) female with diabetes, hypertension, hyperlipidaemia, and a significant family history of cardiovascular disease presented with limiting shortness of breath (winded when performing any activity), worsening over the past few months, as well as atypical chest pain (ache and pressure, sometimes on exertion). The patient also reported recurring syncopal episodes. She underwent nuclear stress testing 2 years prior to the present encounter, which was normal. Further, she received a TTE ∼18 months ago, which was unremarkable other than evidence of advanced diastolic dysfunction. POC-CAD was ordered and performed 5 days later, which was test-positive, with a score of 0.14. A CCTA was then performed ∼5 weeks after POC-CAD, showing a calcium score of 606, along with heavy and diffuse calcification in the proximal LAD and heavy RCA calcification (ostial and mid). Further, severe narrowing at the mid-LAD was apparent. Given the multiple progressive issues, and the POC-CAD and CCTA results, ICA was warranted to assess the patient’s coronaries. The catheterization, ∼5 weeks after CCTA (and about 26 months since stress testing), revealed 10%–20% lesions in LAD D1 and the LCX, as well as tandem 90% lesions at the mid- and distal RCA. Stents were placed for each of the RCA lesions.

## Discussion

This case series highlights the challenges in diagnosing CAD, particularly in high-risk patients with ongoing symptoms and negative nuclear stress testing. Balanced ischaemia may obscure significant multi-vessel disease, leading to false-negative results on nuclear stress testing. Specifically, nuclear stress testing has a known deficiency in patients with globally, homogenously reduced perfusion as can occur in multi-vessel disease, presenting as the absence of focal perfusion defects. This effect has been explored previously in thallium scans, with Christian *et al*.^9^ finding that only 29% of patients with three-vessel or left main disease had perfusion abnormalities in all three territories and Martin *et al*.^10^ reporting that 20% of patients with multi-vessel disease had a normal thallium scan. In contrast to SPECT, CCTA and its derivative tests (primarily CT-FFR) can be an effective tool for assessing balanced ischaemia, as the imaging is directed at the coronary artery itself rather than the perfused myocardium.^11^ POC-CAD has a similar rule-out profile for functionally significant CAD as CCTA (sensitivity of 93% and specificity of 53%^12^), as stated by FDA in the POC-CAD 510(k) clearance summary.^13^ However, SPECT remains the predominant test for ischaemia assessment in the USA, with a recent study^14^ reporting that 77% of patients receive a standalone SPECT, in contrast to 2.1% receiving CCTA. Further, almost half of physicians in the study were found to refer more than 90% of patients to SPECT.

One may reasonably question whether the nuclear stress testing truly identified the absence of significant CAD at the time of testing in these cases, and the disease subsequently developed during the period between the nuclear stress testing and ICA. Coronary artery disease is a chronic disease that is believed to progress over the period of many years, with accelerated atherosclerosis mainly occurring in specific cohorts, such as after heart transplant, coronary artery bypass grafting, and percutaneous transluminal coronary angioplasty.^15^ None of the cases described herein belong to these cohorts. Beyond accelerated atherosclerosis, spontaneous atherosclerosis is another pathway for rapid development, which is precipitated by vascular injury in the form of chronic endothelial damage and resultant functional changes, leading to the hallmark lipid accumulation.^15^ Subsequent to the lipid accumulation, plaque development follows the typical progression. Such a rapid non-linear progression between negative nuclear stress resting and subsequent ICA cannot be excluded; for instance, hypothetically, in Case 5, diabetes-related chronic hyperglycaemia may have caused the endothelial damage^16^ that initiated the spontaneous atherosclerosis pathway. However, we believe that the most likely explanation in these cases remains a false-negative nuclear stress test, especially in the presence of the persistent symptomology.

Further, the assessment of disease probability should be integral to the management of patients such as these. All patients exhibited an appropriate probability of CAD (>15%) to warrant non-invasive testing by at least one pre-test probability model (*Table 1*). While the guidelines provide a coarse estimation of disease probability, clinicians must refine that initial probability with further information about the patient and their own clinical judgement.

POC-CAD aided risk stratification in these five cases, prompting further evaluation and appropriate PCI in three of the five cases. The role of alternative diagnostic modalities in these cases is further supported by guidelines emphasizing multi-modality assessment (i.e. calcium scan, CCTA, and even ICA) for symptomatic patients without known CAD, but with normal stress imaging (including nuclear stress testing).^17^ It is possible that earlier use of a test such as POC-CAD would have increased the suspicion for CAD and may have reduced the delay in care that the patients experienced.

## Limitations

POC-CAD demonstrated clinically important utility in these cases since its relatively high sensitivity of 88% compares favourably with SPECT imaging. However, as a rule-out test, its specificity (51%) means false positives remain a consideration, potentially leading to unnecessary invasive procedures. Further large-scale, prospective studies are needed to define ideal clinical usage and to assess cost-effectiveness. Additionally, integration and comparison with other non-invasive modalities (such as coronary CT angiography, calcium scoring, PET, stress cardiac MRI, and stress TTE) may further characterize and enhance diagnostic performance.

## Conclusion

Balanced ischaemia can mask significant CAD on nuclear stress testing, leading to delayed or missed diagnoses. POC-CAD noninvasively analyses electrical and haemodynamic signals to generate a rapid score for CAD. When used alongside conventional imaging and clinical assessment, it identifies high-risk patients who might otherwise be overlooked. Integrating POC-CAD into diagnostic pathways might reduce delays, guide timely interventions, and improve patient outcomes.

## Lead author biography


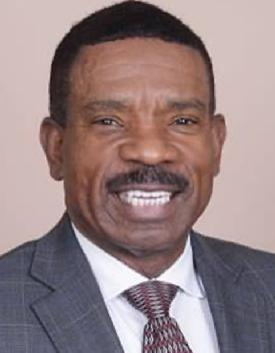


Dr Charles R. Bridges was a Professor of Surgery at the University of Pennsylvania and Chief of Cardiac Surgery at Pennsylvania Hospital. He was Chief Technology Officer for the Cardiovascular and Pulmonary Therapeutic Areas at Janssen Pharmaceuticals (Johnson & Johnson). In 2022, he was elected to the US National Academy of Engineering. Currently, he is CorVista Health Chief Scientific Officer, where his work led to FDA Breakthrough Designation for pulmonary hypertension (PH) and FDA clearance of the CAD and PH diagnostics. He received his AB in Engineering and Applied Physics from Harvard, entering Harvard Medical School at the age of 18. He earned a SM in Electrical Engineering and Computer Science and a ScD in Chemical Engineering from MIT.

## Supplementary Material

ytag016_Supplementary_Data

## Data Availability

The data underlying this article will be shared on reasonable request to the corresponding author.
